# Voices from Hiding: Assessing the Impact of the 2021 Coup on Nursing in Myanmar

**DOI:** 10.1111/inr.70103

**Published:** 2025-09-18

**Authors:** Marcus Wootton, Moh Moh Lwin, San San Oo, Judith Ellis

**Affiliations:** ^1^ International Academy Royal College of Nursing and Honorary Fellow University of Edinburgh Edinburgh UK; ^2^ Independent Nursing Consultant Yangon Myanmar; ^3^ Paediatrician Glangwili Hospital Wales UK; ^4^ Emeritus Professor and Royal College of Nursing Fellow Preston England

**Keywords:** Myanmar nursing, nursing in conflict, nursing education, professional resilience

## Abstract

**Aim:**

This study explores the initial impact of the 2021 Military coup on Myanmar's nursing profession, clinical practice and clinical education.

**Background:**

Myanmar is a country historically riven by conflict; however, between 2010 and 2021, there was democratic reform and partial peace, prompting improvements in nursing practice and health outcomes. This study examines the impact of the 2021 coup, which effectively halted this progress, on Myanmar's nursing. It contributes to the limited literature exploring the effects of civil unrest and governmental coups on nursing, particularly in closed societies such as Myanmar.

**Methods:**

We present a qualitative synthesis of semi‐structured interviews, undertaken with seventeen nurses in Myanmar via secure video link.

**Results:**

Nurses describe the challenges to nursing practice before the coup, the relative progress that Myanmar's nurses made in the short years of quasi‐civilian government and, following the coup, the significant deterioration in working locations, conditions and personal safety for nurses who refused to work in Junta‐controlled hospitals.

**Discussion:**

The coup has damaged all aspects of nursing in Myanmar. These results document a rapid unravelling of progress, accentuated by long‐standing underlying weaknesses in systems and leadership, with additional threats to personal safety. This trend has been previously reported in other conflicts but rarely directly from nurses and never from Myanmar.

**Implications for nursing:**

The coup has been catastrophic for nursing in Myanmar. Participants describe widespread health system collapse, deliberate targeting by the junta and a need for international support.

## 1 Introduction

There is minimal evidence exploring the detailed impact of civil unrest on the nursing profession in any country. Research focuses on the negative implications of unrest for health outcomes (Kloos 2019) and the damage caused to medical rather than nursing education (Sadhaan et al. 2022). What literature indicates broadly is that civil conflict has a devastating impact on nurses and nursing porfession (Rahimaghaee et al. 2016). We set out to document the impact of the 2021 Myanmar coup on the nursing profession, education and clinical practice.

Civil conflict shatters health systems (Sabes‐Figuera et al. 2012), with focus often drawn to the damage caused to frontline curative health infrastructure (Dobiesz et al. 2022a) and limited attention to the impact on workforce development.

Conflict causes surges in health care demand and enormous logistical challenges (Haar et al. 2021). Nurses find themselves at the crisis epicentre and, increasingly, directly targeted (Patel et al. 2016). Garfield and McCarthy (2005) describe the impact of political instability as devastating for nurses in practice. Still, there is limited evidence of the effect of conflict on health workforce development and specifically on nursing education (Dobiesz et al. 2022b).

Following independence from the British, Myanmar was governed by a series of Military Junta (Myoe et al. 2018 and Taylor 2007). Democratic reform began in 2010, and in 2015, the National League for Democracy (NLD), led by Aung San Suu Kyi, was elected to power, taking partial control of the country (Jupe 2023). This period of quasi‐democratic rule saw nursing quantity and capacity increase significantly (Latt et al. 2016), with nurses per 1000 population rising by 28% from 2005 to 2019 (World Bank 2019).

## 2 Background

Myanmar's professional organisations forged strong international partnerships, developing the role of Myanmar nurses from ‘competent professionals’ able to safely deliver care as stipulated by a medical doctor, to ‘experts’ with the knowledge and skills for independent clinical decision‐making and unsupervised practice (Oguro et al. 2022). This progress was to be tragically short‐lived.

On 8th November 2020, the NLD won a democratic election (Renshaw and Lidauer 2021); however, on 1st February 2021, Myanmar's army (junta) staged a coup. Professionals, including doctors and nurses, led marches and boycotted work in junta‐administered hospitals (Chen et al. 2023). Myanmar's Civil Disobedience Movement (CDM) was born.

Since 2021, CDM nurses have worked with medics, setting up a parallel health system and treating patients outside the junta‐controlled hospitals (Than et al. 2024). This ‘ad hoc’ health provision includes ‘pop‐up’ clinics, secret hospitals and outreach to internally displaced people. As a result of direct targeting, CDM nurses have been imprisoned, and many are in hiding (Haar et al. 2024).

## 3 Aim of the study

This study explores the impact of the 2021 coup on nursing in Myanmar. We explore Myanmar nurses’ perceptions of the ‘pre’ and ‘post’ coup profession, clinical practice and clinical education.

## 4 Research Design

We adopted an interpretive phenomenological approach towards understanding participants’ views of their professional roles, identities and ethical challenges. We employed a social research stance during thematic content analysis (Gilbert and Stoneman 2015; May and Perry 2022), also drawing on qualitative research for education (Bogdan and Biklen 1997).

We chose a semi‐structured interview design, based on the principles of effective qualitative interviewing advocated by Kallio et al. (2016). Interviews took place between March and December 2021.

Semi‐structured interviews have been used effectively in qualitative research in Myanmar (Kaji et al. 2015) and provided a practical and ethical method to understand complex phenomena via virtual interviews, ensuring participant safety. Security concerns precluded focus groups; therefore, we conducted one‐to‐one interviews.

We selected this methodology to provide focused responses while offering the flexibility to explore sensitive and dynamic issues (Adeoye‐Olatunde and Olenik 2021). This methodological approach was particularly important for the ‘post‐coup’ element of the study, where, in contrast to the pre‐coup period, the UK‐based research team had no first‐hand experience due to travel restrictions.

This study was designed and reported in alignment with the Consolidated Criteria for Reporting Qualitative Research (COREQ) checklist (Torres‐Gordillo and Rodríguez‐Santero 2023), which provides a framework for transparency and rigour in qualitative research. COREQ guided the reporting of study context, researcher reflexivity, data collection methods and analytical processes to enhance credibility, dependability and transferability.

A reflexivity approach to data analysis supported the phenomenological lens by accepting critical subjectivity (Judd et al. 1991), whereby participants’ thoughts, beliefs and attitudes were viewed not as biases but as valid expressions of meaning (Heron and Reason 1997). This orientation recognised the co‐constructed nature of knowledge and allowed for deeper insight into the emotional and political dimensions of the nurses' experiences (Bourdieu 2020).

We applied inclusive coding (Pfaffenberger 1988) to interlink units of meaning across narratives. Open codes were initially identified and developed into more specific and exogenous categories, with all transcripts coded to explore the generality and significance of recurring patterns (Bryman 2003).

To confirm the reliability and consistency of coding, an independent researcher reviewed the coding and the selection of quotes to illustrate points made, ensuring they reflected indicative data and not just exotic, dramatic data.

### 4.1 Sample and Setting

We recruited a convenience sample of seventeen CDM nurses who were currently in hiding within Myanmar.

### 4.2 Inclusion Criteria

- Registered nurses (or within days of nursing degree completion) in Myanmar at the time of the 2021 coup.
- Self‐reported participation in the CDM.
- Involvement in nursing education both before and after the coup.
- Ability to participate in a virtual interview conducted in Burmese.

### 4.3 Exclusion Criteria

- Individuals inactive in nursing.
- Nurses who were employed in military‐controlled hospitals after the coup or who reported links to the junta.
- Participants who were unable to provide informed consent.
- Individuals without sufficient internet access or a security situation that precluded safe participation.

### 4.4 Participants

Sixteen of the participants stated they were qualified nurses, with two having completed a Master's degree. The 17th participant was undertaking their final undergraduate exam when the junta closed all nursing schools.

The median career duration for qualified participants was four years, ranging from 1 to 28 years. The nurses were all female, and pre‐coup had been working in a wide range of hospital inpatient areas.

Participants’ real names were never collected, during virtual interviews, video was disabled, and nurses and researchers used unique IDs, aliases and password‐protected, locked meetings.

All nurses confirmed that participation posed no greater personal risk.

One‐to‐one semi‐structured interviews were conducted virtually by a UK doctor or nurse and a Burmese‐speaking clinician. The open questions (see Table 1) were asked universally, with follow‐up probing where clarification was needed.

**TABLE 1:** Questions.

1	Are you a qualified registered nurse?
2	How many years have you been working as a nurse?
3	Which area of nursing were you working in before the coup?
4	What elements of nursing did you enjoy or find satisfying before the coup?
5	What did you not enjoy or find challenging about working as a nurse before the coup?
6	Have you been working since the coup? If yes: doing what?
7	What has been difficult since the coup?
8	What pre‐coup experience and training have been useful for your practice since the coup?
9	What experience and changes to nurse training would be useful in the future?
10	Anything else that you would like to raise?

Interviews, conducted in Burmese, were recorded securely, transcribed verbatim, anonymised and translated into English by three UK‐based Burmese medical colleagues. Recordings were then deleted, and translators aimed for faithful translation, with only necessary sentence adjustments to preserve meaning.

We used the English language transcripts for thematic content analysis. Major and sub‐themes were identified (see Table 2 for major themes), with supportive verbatim quotes attached. The quote location was noted to provide context during the write‐up.

**TABLE 2:** Major themes summarised.

Theme	Pre‐Coup	Post‐Coup	Future
**The health system of Myanmar**	Gradual improvements; still challenges, including leadership.	In disarray; care given in secrecy; shortages, unsafe conditions.	Hope for restoration once stability is regained.
**Nursing as a profession**	Mixed feelings: pride vs. regret, weak regulation, blurred roles.	CDM nurses banned from work; no income; volunteering; disillusionment.	Some may abandon profession; others vow to return post‐dictatorship.
**Clinical nursing**	Overwork, role confusion, non‐nursing tasks, poor student education.	Unsafe; underground trauma care; constant fear, mental health struggles.	Dream of resuming care under better conditions.
**Nursing education in practice**	Theory–practice gap; students mistreated on placements.	All programmes stopped; some offer risky community education during COVID.	Education seen as a priority post‐recovery.
**Nurses’ view of public perception of nursing**	Some appreciation; others faced misunderstanding and blame.	Nurses feel guilt, isolation, unable to serve openly.	Hopes to rebuild public trust through reform and service.

Subsequently, we organised the principal themes into the sections outlined below:

- The health system of Myanmar
- Nursing as a profession
- Clinical nursing
- Nursing education in practice
- Nurses’ view of public perception of nursing

### 4.5 Ethical Approval

Due to the participants’ actions resisting the Military junta that controlled the Ministry of Health and Sports, seeking ethical approval through official channels was not feasible. Instead, strict ethical standards were upheld and carefully reviewed by post‐doctoral researchers from both the UK and Myanmar, all with nursing backgrounds.

Emphasis was placed on ensuring participant safety, maintaining confidentiality and securing informed consent. We used the UK National Health Service Medical Research Council to guide the principles of this educational evaluation (Medical Research Council 2024).

No patients were contacted during this study; the nurses remain anonymous, and findings are not intended to be generalised. The authors accept these limitations but emphasise the critical value of amplifying the voices of Myanmar's nurses.

Informed consent was obtained with great care from each CDM nurse who agreed to take part. Interview questions (Table 1) were shared with participants in advance, and their understanding and agreement were confirmed before proceeding.

## 5 Findings

Interview questions started with a focus on nurses’ perceptions of nursing in Myanmar before the coup to understand the pre‐existing health landscape.

### 5.1 Pre‐Coup

To protect anonymity, all participants will be quoted by participant numbers (P).

#### 5.1.1 The Health System of Myanmar

All 17 nurses described a preceding decade of health system improvement. This was summarised as ‘Health care being improved and modernised greatly of late’ (P9). Despite the overall positive trajectory, there was a feeling that crucial quality improvement remained undone.

#### 5.1.2 Nursing as a Profession

Some participants expressed pride in nursing: ‘I am happy with my profession’ (P11) and ‘I chose nursing as a career as it is a noble profession’ (P9). One participant implored, ‘future trainees to choose nursing only if you really love the profession, otherwise, you will regret it and bad for patients too’ (P8).

Not all felt professional pride before the coup. One nurse stated they:

Regretted being a nurse in Burma. The role of nurses is disappearing, only handful of people trying to improve, but their efforts are in vain due to lack of vision from seniors. Although nursing education is better, public underestimate nurses' role. Wonder if things could be improved in my lifetime. (P11)

Regulation was identified as a weakness, with one participant stating: ‘There should be a professional body and legal system to prevent the confusion and fake nurses, to protect nursing profession’ (P2).

Participants suggested a ‘need (for) clearly defined roles and responsibilities for staff’ (P2); one identified ‘not having well‐defined job descriptions’ (P8).

Nurses identified public confusion between nurses and nursing aids. For example, participants stated that ‘There are so many training centres for nurse aids. They wear similar uniform, doing invasive procedures, confusing general public’ (P2) and ‘many training schools for nurse aids. No proper uniform policy, hence, nurse aids pretending to be a nurse. Poorly educated general public confused between real nurses and nurse aids’ (P5).

Additionally, there were calls for ‘well‐defined roles between nurses and doctors’ (P15) as the ‘role of nursing colleagues is conflicting with doctors’ (P11). A nurse stated that pre‐coup nurses were **‘**having to see doctors as senior staff’ (P7). One nurse, in considering relationships, stated:

If there is any conflicts between nurses and doctors/nurses and patients' attendants, there should be a proper support system for juniors. All staffs both seniors and juniors should work together to improve the health care system. Build mutual respect, educating general public, lessening the abuses will alleviate the extra stress at work. (P5)

Employment conditions were identified as necessary, particularly in relation to pay. One nurse saw nursing as a ‘respectable profession with regular income’ (P2), with another stating, ‘Although my salary is low, I was very happy and contented’ (P9).

Six participants were not so accepting of low pay. One stated ‘Low pay’ and ‘lack of accommodation’ as the ‘major challenges leading to disillusioned’ and ‘demoralised’ nursing staff (P14).

Leaders were an essential element when considering pre‐coup nursing. Participants expressed satisfaction when ‘working under a leader who put people first; whom people trusted’ (P9), and ‘working with senior colleagues who listen, appreciate, are supportive of my decisions and teach me pros and cons of each’ (P12).

It was seen as essential to be ‘warmly welcomed by seniors, seniors who take responsibilities’ (P7), and seniors who teach things ‘which I hadn't learned at Uni days’ (P5).

Complaints about nursing leadership were also raised, including ‘lost opportunities due to lack of leadership from seniors’ (P6); other nurses expressed the view that there was ‘no‐good leadership among nursing profession’ (P14) with ‘seniors who are biased and promote favouritism’ (P13), unsupportive of some nurses who ‘got blamed for giving proper nursing care’ (P7).

A participant stated how important it was that ‘senior nurses warmly welcoming trainees, having mutual respect…protecting trainees from abuses’ (P2).

One nurse felt ‘The role of nurses is disappearing, only handful of people trying to improve, but their efforts are in vain due to lack of vision from seniors’ (P11).

#### 5.1.3 Clinical Nursing

There was general agreement that the greatest pre‐coup challenge was a ‘heavy workload due to a lack of human resources’ (P13) and a ‘shortage of nursing staff unable to give proper nursing care that patients deserved’ (P1). One participant said, ‘If I can provide proper nursing care, will be much happier and satisfied at work’ (P9).

They described ‘so many nursing vacancies misbalancing nursing patient's ratio’ (P4), and an ‘excessive workload making it impossible to provide the care that patients deserve made me feel dissatisfied’ (P1). Staff shortages also impacted attendance at pre‐coup training courses. A participant stated that students were ‘unable to take part in discussions and learn more from interesting cases due to constant tiredness from heavy workload’ (P13).

One participant stated there was a ‘heavy workload, lack of trust by patients, mutual respect, co‐operation … between colleagues created stressful working environment; break down professional relationship and dissatisfied at work’ (P9).

Expectations to undertake inappropriate tasks exacerbated the workforce issues. A participant stated, ‘unrealistic expectations by seniors for us to undertake non‐nursing tasks make me unhappy’ (P5). Examples given were ‘unnecessary paper works’ (P4), ‘occasionally having to do chores for doctors, nothing to do with nursing’ (P4) and ‘having to boil water for professor and cleaning the room’ (P3).

One nurse summarised ‘having to do job not related to nursing (is) missing out on patients care’ (P15). Another patient felt ‘Juniors should uphold the high nursing role, able to say no without fear to non‐nursing unnecessary work and concentrate on patients’ care. We the nurses must unite and help each other’ (P16).

#### 5.1.4 Nursing Education in Practice

Participants raised repeated concerns of a ‘huge gap between theoretical knowledge and practical skills’ (P14). One nurse stated there ‘should be balanced theoretical and practical training’ (P2) and student nurses should ‘read widely and apply knowledge into practice’ (P4). A nurse suggested that ‘Practical skill should be taught by seniors effectively during hospital placement, enabling the trainees to work with confidence, equipped with knowledge and skills’ (P5).

There were consistently negative descriptions of pre‐coup clinical training. One participant stated, ‘During hospital placements, we were not taught practical skills but treated as unwelcomed nuisance extra‐bodies, had to do dusting of beds, moving beds around, scrubbing floor, cleaning windows; these tasks are not compatible for nursing training’ (P2).

Others suggested, ‘juniors should learn not only routine but diseases also during ward placement’ (P3), ‘Concentrate or think about improving necessary nursing care’ (P4) and accept the nurses’ role in ‘health education of patients and carers’ (P2).

There was a universal view that ‘nursing roles and responsibilities need to be clearly defined’ (P7) and ‘not just seen as theoretical’ (P7).

#### 5.1.5 Nurses’ View of Public Perception of Nursing

The gratitude of patients was central to six participants’ satisfaction, who felt respected when ‘caring for patients who appreciate and understand our work’ (P12), and when ‘patients appreciated and thanked our work’ (P14). One said ‘satisfied that [they were] able to educate patients’ (P15).

This positivity was counterbalanced by others who described ‘undisciplined patients’ (P15), ‘dealing with patients and their carers who do not understand the importance of a disciplined approach’ (P12) and that the ‘[p]ublic underestimate nurses’ role’ (P11).

Some described ‘misunderstandings’ (P14) and ‘tempers flaring’ (P14) when there were ‘long waiting times’ (P13) or when ‘not enough medicine. Having to ask patients to buy, causing extra financial burden for the poor’ (P4).

The respondents felt that patients viewed nurses as ‘having to take sole responsib[ility] for drinking water supply, drugs supply, maintenance of surgical instruments’ (P6), difficulty with the ‘corruption’ (P14) that occurred pre‐coup.

### 5.2 Post‐Coup

The interviews progressed to the post‐coup situation.

#### 5.2.1 The Health System of Myanmar

All 17 nurses described a health system in disarray. The CDM nurses faced unprecedented risk and repeatedly identified challenges ‘to give care with limited resources’ (P14). One nurse described ‘at IDP camp, no reliable phone, internet connection, electricity supply, hindrance to give health care. Unable to get necessary medical supply’ (P4). A nurse clarified ‘medical care had to be done underground, even donating medicine’ (P17). Another participant described her frustration that ‘My experience and skills are useful, but unable to get access to necessary instruments quickly, even buying one must be in secret’ (P17).

#### 5.2.2 Nursing as a Profession

The nurses discussed their current practice. The military, according to the interviews, had banned CDM staff from state and private hospitals. Quotes include ‘private sectors do not hire CDM staff and even sacked the existing staff who are on CDM’ (P2). None of the nurses interviewed had income. Some cited a loss of independence, one saying, ‘I am back living with my parents helping them out. Current challenges are not having regular income, relying on my parents’ (P4). Another stated: ‘I am unable [to] earn money as I am in hiding’ (P16).

Some worked voluntarily, including ‘giving health education via social media from time to time’ (P1) and providing local community care. One stated:

After the military coup, I am not willing to work under dictatorship. Hence, I went back to my native place. I am giving health care in my community as much as I could and also answering health‐related questions via Facebook messenger in collaborating with doctor friend to solve health problems, COVID prevention and treatment. (P5)

A nurse stated that she ‘currently (had) no feeling towards nursing profession. Injustice, oppression, no defined role, responsibilities, and no principles make me feel numb. There's a lot to reform’ (P10). This sentiment revisited pre‐coup frustrations of unrecognised qualifications. One nurse stated, ‘although I finished a Master degree, there is no prospect of career progression. I am same level as the one with diploma. That system should change’ (P3).

#### 5.2.3 Clinical Nursing

The dangers the nurses faced in the months following the coup are all too evident, and none of the seventeen participants were consistently safe to work. One stated that she is ‘currently living in fear for my freedom and unable to provide health care, making me discontent’ (P1).

One nurse said she was ‘[n]ot willing to work under military dictatorship’ and ‘joined CDM medical cover team for the protestors soon after coup, giving health care for the community. Due to risk of arrest and detention, unable to travel freely’ (P5).

All the participants mentioned feeling unsafe or ‘hiding’ (P16). They vividly shared the ‘scare of firing noises from RPGs [Rocket Propelled Grenades]’ (P15).

Other nurses outlined the clinical practice which they had undertaken during the conflict. Universally, this had shifted from state‐run health hospitals to pre‐hospital care in mobile and ‘pop‐up’ clinics. One nurse stated, ‘experience enabling to give emergency care (e.g. treating injury due to RPG, hypovolemic shock, Diabetes Mellitus)’ (P4). As one nurse shared, ‘due to shooting, I have to give emergency treatment and also follow protesters for medical cover’ (P17).

Other responses referred to being ‘able to use my experience and skill to care for family member and neighbourhood’ (P16) and ‘useful when family members get unwell’ (P14). COVID was frequently referred to with experience and skills enabling nurses ‘to look after COVID patients at home’ (P10) and being ‘able to give advice to COVID suspected cases in my neighbourhood’ (P13).

The nurses also described providing emergency care during violence. One nurse said:

Due to shooting I have to give emergency treatment and also follow protesters for medical cover. Sometimes the soldiers know of wounded person getting treatment coming after us, we have to run carrying the wounded and splitting the group. Sometimes they are after dead bodies, so I have to hide the dead body and run for my life; sometimes I am unable to provide care for fear of my personal safety. Medical care had to be done underground. (P17)

Participants described practising as volunteers ‘underground in secret’ (P3). One nurse stated:

During my stay at the IDP (Internally Displaced People) camp, I cared for injured patients who were engaged in the CDM who were gravely ill. I was able to help them by giving emergency treatment, health education and also consultation with doctors by phone for management. (P4)

They described ‘military dictators arresting the staff in the CDM’ (P5), how they had ‘sacrificed everything as CDM nurse, living like fugitive, constant fear of my personal safety’ (P9) and how ‘when there is news of rounding CDM up and arrest, have to stay away from my home and hide somewhere’ (P1). Fear was not limited to nurses’ personal safety but also ‘fear for safety of family’ (P2) with ‘military dictators arresting the staff in CDM and their families’ (P5). They also shared their ‘low self‐esteem’ (P2) due to their ‘inability to provide for family’ (P2).

One participant stated:

After military take over, I am jobless, sleeping at different hideout with my small backpack. I am living and moving around in secrecy, however, need to get back home from time to time to see to my elderly mother and family, a risk of arrest and detention. (P17)

One nurse described the isolation from ‘my team which is my family whom I'm missing dearly’ (P11), and another described having ‘cut off friendship with the people who became non‐CDM … sad for that’ (P6).

One nurse stated that the ‘military coup make[s] it intolerable to stay at work and be a nurse’ (P2).

‘Feeling sad and depressed’ (P3) was mentioned by many. One nurse stated, ‘As an ordinary citizen, I feel insecure and miserable. Experiencing a profound sense of loss and despair due to atrocities committed by the military junta, day after day, affecting my morale and mental health’ (P6).

A nurse shared that ‘currently, as a nurse, I am under so much psychological pressure’ (P2). Another explained that ‘now the whole country is suffering, unable [to] give health care, sadden(s) me’ (P9). This statement was reinforced by another nurse who said, ‘I am depressed, being unable to serve the people, affecting me psychologically. I wish to end the deaths and dying from COVID‐19 and civil war’ (P16).

#### 5.2.4 Nursing Education in Practice

All 17 nurses reported that all educational programmes had effectively stopped since the coup. COVID had surged in May 2021 and became a focus for many nurses. Recognising the acute need, but unable to provide ‘in‐person’ (P1) care, many nurses had pivoted to virtual public‐facing health education, at considerable risk to personal safety.

Participants answers included, ‘I did lots of teleconsultation for COVID patients, received huge phone bill’ (P17) and ‘I do provide health education and care in my community, specially to control and treat COVID outbreak along with one other doctor and post‐COVID care, including education on nutrition with the best of my ability’ (P2).

#### 5.2.5 Nurses’ View of Public Perception of Nursing

A nurse described being ‘unable to serve the community and help general public due to concern of personal safety making me feel guilty, self‐blaming, full of regrets for letting down deceased and detained friends’ (P6).

The nurses interviewed were unwilling to articulate their view of the current public perception of nursing. Still, guilt was a recurrent theme, including being ‘Not content with what I've done. Unable to do openly and bravely what needs doing’ (P7), ‘unable to make use of my skills as I am in hiding’ (P15).

One participant summarised, ‘I constantly fear for my safety, unable to help IDPs as much as I would like to, unable to co‐ordinate and cooperate due to COVID travel restrictions’ (P4).

## 6 Future

The future was universally identified as uncertain, with no foreseeable employment for CDM nurses and feelings of ‘abandoning the profession’ (P5). One nurse stated, ‘I am so bitter for losing my freedom and chances for my futures and living like fugitive’ (P9).

The final comments made were aimed at ending the coup. Statements included, ‘We need to overthrow the junta, stand with people, must fight just war together. Won't rest till we win’ (P3), ‘We must resist and overthrown illegal take‐over of power. We must try together to win the just war. We won't rest’ (P9), ‘we can't live under military, we must win, we must explore all ventures to make a change’ (P8).

A nurse described how she ‘sacrificed everything as CDM nurse, living like fugitive, constant fear of my personal safety, unable to provide health care while the public needed most, with that resentment I swear to fight till we win’ (P5). A nurse reflected that, ‘It'll make life much easier if unlawful arrest and arbitrary detention stopped. Life will be transformed once the people get freedom and regain human rights’ (P13).

The nurses saw ‘It is very important to overthrow the dictatorship quickly’ (P16) and ‘Once we overthrown the dictatorship, won the revolution war, I'll go back to my work’ (P16).

There was final positivity for the future with statements that, ‘Once we have our country back in a stable state and COVID gone, everything will be fine’ (P4), with a plan expressed that, ‘Once the country established peace and stability, first thing to do is health care, second is education’ (P5), but this would require nurses ‘to have patience and innovative thinking to compensate poor health care system’ (P5).

One nurse stated, ‘Justice will prevail one day. I believe and believe in it’ (P6).

## 7 Discussion

We have presented an analysis of the impact of the 2021 coup on Myanmar's nursing profession and nurse education.

The lived experience of the nurses interviewed indicates that the decade of fragile progress has been reversed and arguably made worse by Myanmar's coup.

Participants described significant improvements during the pre‐coup years, including the expansion of nursing education, increased workforce capacity and efforts towards professionalisation.

However, this progress was undermined by structural weaknesses such as unclear role definitions, insufficient leadership support and an imbalance between theory and practice in clinical education. This proved detrimental to the resilience of Myanmar's nursing profession when the coup came.

Since the coup, the professional lives of nurses have been radically disrupted beyond recognition. The transition from formal paid employment as nurses in hospital‐based practice to volunteer care in community or conflict settings, often under threat of arrest or violence, has led to extreme professional, psychological and ethical strain.

Many participants expressed grief, fear and guilt, having lost both professional standing and personal security. Nevertheless, their ongoing contributions to COVID‐19 care, trauma support and health education demonstrate extraordinary resilience and commitment to nursing.

These findings echo global literature on the vulnerability of health systems in conflict zones (Ugwu et al. 2025; Ekzayez et al. 2021) but uniquely foreground the voices of nurses operating in authoritarian Myanmar.

## 8 Limitations

We conducted semi‐structured interviews with a convenience sample, all of whom were female nurses affiliated with the CDM. This excluded male nurses and those still employed in junta‐controlled institutions, limiting broader applicability.

Our design allowed for safe, ethical data collection; however, the remote interviews may have limited interaction depth. We were unable to conduct follow‐up interviews or response checking, which may have affected the validity of our interpretation.

Translation from Burmese to English risks losing linguistic and cultural nuance, particularly between such different societies. We selected manual coding to capture narrative richness, though it may introduce subjectivity and reduce reproducibility compared to computer‐assisted qualitative analysis.

For security, anonymity protocols meant demographic and geographic details were not recorded, reducing contextual depth. Combined with the small sample, this means the voices in this study cannot be assumed to reflect the experiences of all Myanmar nurses.

Despite these limitations, the study offers rare insights into a unique crisis.

## 9 Conclusion

This study highlights how the 2021 military coup in Myanmar has devastated the nursing profession, reversing a decade of fragile progress and exposing the vulnerability of healthcare systems in politically unstable contexts. The findings demonstrate the severe consequences for clinical practice, education, and the personal safety of nurses, particularly those resisting junta control. By documenting these experiences directly from frontline nurses, this research adds a rare and essential perspective to the global evidence base on the effects of conflict and authoritarian rule on nursing. Urgent international solidarity, advocacy, and targeted support are needed to sustain Myanmar's nurses, protect their safety, and preserve the capacity of the profession to recover when political conditions allow.

## 10 Implications for Nursing

The testimonies identify international partnerships, including those established pre‐coup, as critical for building resilience. These should be encouraged, funded and developed. Programmes should focus on leadership and systems development to buttress the health service in a future crisis.

This analysis documents the extraordinary pressure nurses, who chose not to work for junta‐controlled health services, came under and the risks to their personal safety. There is an urgent need for the global community to strengthen protections for nurses working in politically unstable settings.

This includes prosecutions for those who target nurses, supporting alternative education models and establishing safe channels for professional engagement.

The experiences of Myanmar's nurses remind us that during times of collapse, it is nurses who persist, quietly and courageously holding together the threads of health, care and dignity.

## Author Contributions

Evaluation design: MW and JE. Data collection: MW, SSO and JE. Data analysis: MW, MML, SSO and JE. Evaluation supervision: MW. Manuscript writing: MW, MML, SSO and JE. Critical revisions important to context: MW and MML. Translation: MML and SSO.
